# Outcomes of Coronavirus Disease 2019 (COVID-19) Related Hospitalization Among People With Human Immunodeficiency Virus (HIV) in the ISARIC World Health Organization (WHO) Clinical Characterization Protocol (UK): A Prospective Observational Study

**DOI:** 10.1093/cid/ciaa1605

**Published:** 2020-10-23

**Authors:** Anna Maria Geretti, Alexander J Stockdale, Sophie H Kelly, Muge Cevik, Simon Collins, Laura Waters, Giovanni Villa, Annemarie Docherty, Ewen M Harrison, Lance Turtle, Peter J M Openshaw, J Kenneth Baillie, Caroline A Sabin, Malcolm G Semple

**Affiliations:** 1 National Institute for Health Research (NIHR) Health Protection Research Unit (HPRU) in Emerging and Zoonotic Infections, Institute of Infection, Veterinary and Ecological Sciences; Faculty of Health and Life Sciences, University of Liverpool, Liverpool, United Kingdom; 2 Liverpool University Hospitals National Health Service (NHS) Foundation Trust, member of Liverpool Health Partners, Liverpool, United Kingdom; 3 Division of Infection and Global Health Research, School of Medicine, University of St Andrews, St Andrews, United Kingdom; 4 HIV i-Base, London, United Kingdom; 5 Mortimer Market Centre, Central and North West London NHS Foundation Trust, London, United Kingdom; 6 British HIV Association, London, United Kingdom; 7 Department of Global Health and Infection, Brighton and Sussex Medical School, University of Sussex, Brighton, United Kingdom; 8 Centre for Medical Informatics, Usher Institute, University of Edinburgh, Edinburgh, United Kingdom; 9 Intensive Care Unit, Royal Infirmary Edinburgh, Edinburgh, United Kingdom; 10 National Heart and Lung Institute, Imperial College London, London, United Kingdom; 11 Roslin Institute, University of Edinburgh, Edinburgh, United Kingdom; 12 University College London (UCL), London, United Kingdom; 13 NIHR HPRU in Blood Borne and Sexually Transmitted Infections at UCL, London, United Kingdom; 14 Respiratory Medicine, Institute in The Park, University of Liverpool, Alder Hey Children’s Hospital, Liverpool, United Kingdom

**Keywords:** COVID-19, SARS-CoV-2, HIV, mortality

## Abstract

**Background:**

Evidence is conflicting about how human immunodeficiency virus (HIV) modulates coronavirus disease 2019 (COVID-19). We compared the presentation characteristics and outcomes of adults with and without HIV who were hospitalized with COVID-19 at 207 centers across the United Kingdom and whose data were prospectively captured by the International Severe Acute Respiratory and Emerging Infection Consortium (ISARIC) World Health Organization (WHO) Clinical Characterization Protocol (CCP) study.

**Methods:**

We used Kaplan-Meier methods and Cox regression to describe the association between HIV status and day-28 mortality, after separate adjustment for sex, ethnicity, age, hospital acquisition of COVID-19 (definite hospital acquisition excluded), presentation date, 10 individual comorbidities, and disease severity at presentation (as defined by hypoxia or oxygen therapy).

**Results:**

Among 47 592 patients, 122 (0.26%) had confirmed HIV infection, and 112/122 (91.8%) had a record of antiretroviral therapy. At presentation, HIV-positive people were younger (median 56 vs 74 years; *P* < .001) and had fewer comorbidities, more systemic symptoms and higher lymphocyte counts and C-reactive protein levels. The cumulative day-28 mortality was similar in the HIV-positive versus HIV-negative groups (26.7% vs. 32.1%; *P* = .16), but in those under 60 years of age HIV-positive status was associated with increased mortality (21.3% vs. 9.6%; *P* < .001 [log-rank test]). Mortality was higher among people with HIV after adjusting for age (adjusted hazard ratio [aHR] 1.47, 95% confidence interval [CI] 1.01–2.14; *P* = .05), and the association persisted after adjusting for the other variables (aHR 1.69; 95% CI 1.15–2.48; *P* = .008) and when restricting the analysis to people aged <60 years (aHR 2.87; 95% CI 1.70–4.84; *P* < .001).

**Conclusions:**

HIV-positive status was associated with an increased risk of day-28 mortality among patients hospitalized for COVID-19.

## Teaser

People with human immunodeficiency virus (HIV) hospitalized with coronavirus disease 2019 (COVID-19) had an age-adjusted 47% higher risk of day-28 mortality compared with a large population of HIV-negative people within the same data set. The effect persisted after adjusting for sex, ethnicity, and major comorbidities.

Older age and presence of comorbidities including immunosuppression are recognized to increase the severity of coronavirus disease 2019 (COVID-19) [1–5]. However, existing evidence for an association between human immunodeficiency virus (HIV) infection and COVID-19 related outcomes is mixed. Despite effective antiretroviral therapy (ART), people with HIV (PWH) may continue to experience persistent immunodysfunction [6, 7], which might promote COVID-19 severity, or conversely, attenuate its pathological immune responses [8]. Although some antiretroviral drugs have been proposed to protect against COVID-19, the data remain uncertain [9, 10]. Importantly, the common occurrence of cofactors such as diabetes and chronic renal and pulmonary disease [11], alongside disadvantageous socioeconomic variables [12], may increase the risk of adverse outcomes among PWH who acquire severe acute respiratory syndrome coronavirus 2 (SARS-CoV-2).

Several case series and observational cohort studies have described the outcomes of COVID-19 in PWH across Europe [9, 13–19], Asia [18, 19], and the United States [8, 18–22]. These studies have often been limited by small sample sizes, lack of direct comparative data from people without HIV, or inability to adjust for comorbidities. Some reports indicated that PWH did not experience higher rates of COVID-19 related hospitalization or mortality than people without HIV [14, 21], whereas others suggested increased disease severity [9, 20]. Importantly, preliminary data from South Africa indicated that despite effective ART, HIV infection more than doubled the risk of COVID-19 related mortality [23].

To characterize the presenting characteristics and outcomes of COVID-19 related hospitalization in PWH relative to those without HIV in the United Kingdom (UK), we analyzed data collected within the International Severe Acute Respiratory and Emerging Infection Consortium (ISARIC) World Health Organization (WHO) Clinical Characterization Protocol (CCP), the largest prospective observational study of patients admitted to hospital with COVID-19 worldwide [24].

## METHODS

### Setting and Participants

The ISARIC WHO CCP-UK is an ongoing prospective cohort study in hospitals in England, Scotland, and Wales [24]. The study was activated on 17 January 2020. The protocol, case report form (CRF) and other study materials, and details of the Independent Data and Material Access Committee are available online [24]. Eligible patients were people aged ≥18 years who were admitted to participating hospitals (207 at the time of analysis) with either laboratory-confirmed or highly likely (based on clinical, laboratory, and radiological findings) SARS CoV-2 infection. Polymerase chain reaction (PCR)-based virus detection was the only test available during the study, and the decision to test was at the discretion of the attending clinical team, who also decided upon hospital admission, transfer into critical care, and use of ventilation.

### Data Collection

Baseline was defined as the date of hospital admission or symptom onset (for those with symptom onset after hospitalization, see below). We included individuals with a baseline date that was on or before 4 June 2020 for whom ≥14 days had elapsed at data extraction on 18 June. Individuals with missing admission date were excluded. Where the date of symptom onset was missing, we assumed that symptoms began on the date of the first SARS-CoV-2 PCR test. Based on the date of symptom onset relative to the date of admission, the infection was classed as community-acquired (symptom onset <3 days), indeterminate (3–7 days), probable hospital-acquired (8–14 days), and definite hospital-acquired (>14 days). Using the CRF version 9.2 [24], demographics, comorbidities, and concomitant medications were recorded on admission; measures of disease severity and laboratory test results were recorded on day 1 (baseline), day 3, day 6, day 9, and on the day of admission to critical care if applicable. CRF-reported HIV-positive status was confirmed via recorded ART, *Pneumocystis jirovecii* prophylaxis in the absence of non-HIV indications (n = 2), or directly with a site investigator. Individuals with missing HIV status and those with unconfirmed HIV-positive status were excluded.

### Statistical Analysis

Presenting characteristics were compared between HIV-positive and HIV-negative people and between PWH who died and those who survived at 28 days using Wilcoxon rank sum tests (for continuous variables) and Pearson χ ^2^ or Fisher exact test (for categorical variables). For all individuals, follow-up ended on the date of death. Patients discharged for home palliative care were considered to have died 3 days afterward. Follow-up was right-censored at day 28 for those remaining alive as an inpatient or for those who were discharged (excepting palliative discharge) prior to day 28. No deaths were recorded among PWH after day 28. Follow-up was censored for patients transferred to another facility at date of transfer; among those with unknown outcome, it was censored on the last recorded date of follow-up where they were known to be alive. For patients who died, were transferred, or discharged on the date of admission or who had no further follow-up recorded beyond the first day, we recorded 0.5 days of follow-up. The primary analysis used a Kaplan-Meier approach to display the cumulative mortality over this period and in strata defined by sex and age. Cox proportional hazards regression with the Efron method for ties was then used to describe the association of mortality with HIV status, before and after adjustment for the following potential confounders: sex, ethnicity, age (in quadratic form), indeterminate/probable hospital acquisition of COVID-19 (as defined above), and 10 separate comorbidities at admission (a series of binary variables to indicate the presence or absence of each of chronic cardiac disease, chronic pulmonary disease, chronic renal disease, diabetes, obesity, chronic neurological disorder, dementia, liver disease [mild, moderate, or severe], malignancy, and chronic hematological disease). These variables were selected a priori. We also included adjustment for the baseline date to account for changes in mortality over time. For partially missing comorbidity data, we assumed missing comorbidities were absent. Participants with completely missing comorbidity data were excluded from these adjusted analyses. We fitted a further model adjusting for disease severity at presentation, defined as oxygen saturation <94% on air or receiving oxygen therapy, to assess whether risk of mortality in PWH could be explained by a different stage of disease advancement at hospitalization. Finally, we repeated the same analysis among individuals aged ≤70 years. A series of sensitivity analyses were performed: (i) we repeated the analyses after censoring follow-up on the day of discharge; (ii) we included those with definite hospital-acquired COVID-19; (iii) we used symptom onset date as the baseline date for all; (iv) we excluded PWH lacking a record of ART; (v) we excluded those without a recorded positive SARS-CoV-2 PCR result; (vi) we calculated propensity scores for HIV-positive status using a logistic regression model based on sex, ethnicity, age (in quadratic form), indeterminate/probable hospital acquisition of COVID-19, smoking status, baseline date, and 10 comorbidities, and included the propensity score in a univariate Cox regression model for death at 28 days; (vii) we considered a binary endpoint of 14-day mortality and performed logistic regression (with the same confounder adjustment as described above); and (viii) we used a competing risks regression model with discharge as a competing risk for mortality. In PWH, we used a Cox proportional hazard model to investigate associations with day-28 mortality. Analyses were conducted in Stata v16.1 (Statacorp, TX, USA).

### Ethical Considerations

Approval was granted by the following Ethics Committees: South Central-Oxford (Ref 13/SC/0149), Scotland (Ref 20/SS/0028), and WHO (RPC571, RPC572). Data collection did not require consent. The study was carried out in accordance with the Helsinki Declaration.

## RESULTS

### Participants

**Figure 1.**
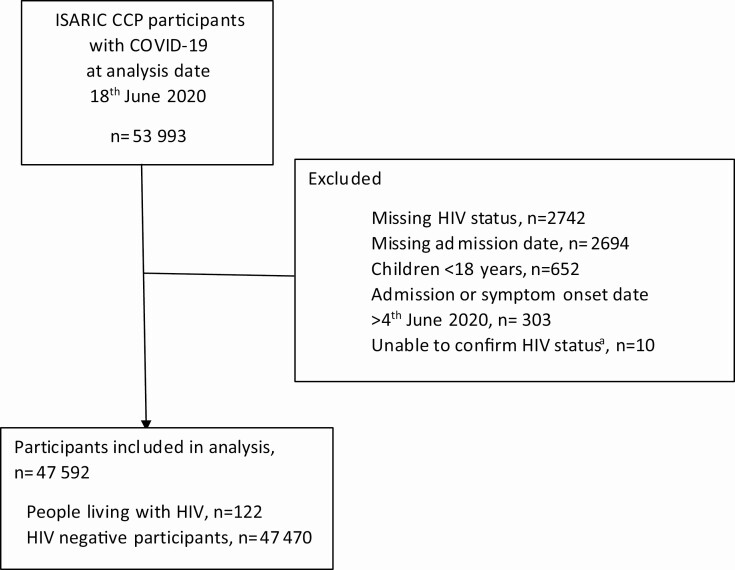
Flowchart of study participants. Abbreviations: CCP, Clinical Characterization Protocol; COVID-19, coronavirus disease 2019; HIV, human immunodeficiency virus; ISARIC, International Severe Acute Respiratory and Emerging Infection Consortium. aHIV status was confirmed if participants were recorded as receiving anti retroviral therapy or prophylaxis against *Pneumocystis jirovecii*, or if local site investigators were able to confirm HIV status.

ISARIC CCP-UK recorded 53 993 people with COVID-19 between 17 January and 18 June 2020. After excluding non-eligible participants (Figure 1), we included 47 592 patients, of whom 122 (0.26%) had confirmed HIV infection. A positive SARS-CoV-2 RNA PCR test was recorded for 43 062/47 592 (90.5%) individuals. Patients excluded from the analysis did not differ by sex, ethnicity, or age; in particular, the characteristics of those excluded due to missing HIV status closely resembled those reported to be HIV-negative (Supplementary Table 1). Among PWH, 1 person was diagnosed with HIV during the admission, and 112 (91.8%) had an ART record. The regional distribution of study participants was similar to the UK population of PWH receiving care (Supplementary Table 2).

### Characteristics at Presentation

**Figure 2.**
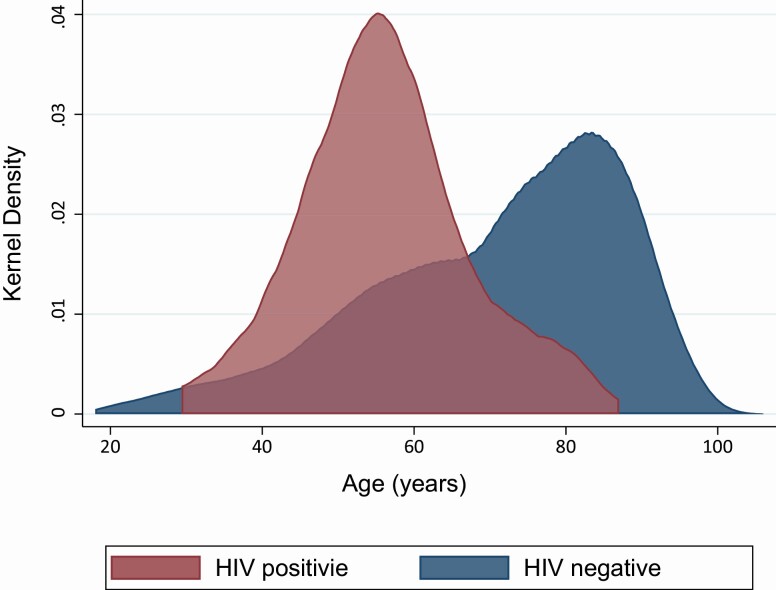
Kernel density plot of age distribution of study participants stratified by HIV status. Abbreviation: HIV, human immunodeficiency virus.

PWH were younger than HIV-negative people (median 56 vs 74 years, *P* < .001) (Table 1, Figure 2) and comprised a greater proportion of males and people of Black ethnicity. PWH had fewer comorbidities overall, and a lower prevalence of cardiac, pulmonary, and rheumatological disease, dementia, and malignancy but higher rates of moderate/severe liver disease; proportions with chronic renal disease, diabetes, and hematological disease were similar. The duration of symptoms was longer in PWH (median 5 vs 3 days, *P* = .002) (Table 2**).** PWH were more likely to present with fever, headache, myalgia, and tachycardia, and to have cough and chest pain. Respiratory rate, occurrence of tachypnoea and hypoxia, and radiological evidence of chest infiltrates did not differ significantly between the 2 groups. PWH presented with lower total white blood cell and platelet count but higher lymphocyte count and C-reactive protein (CRP) (Table 3).

**Table 1.** Summary of Participant Characteristics, Stratified by Human Immunodeficiency Virus (HIV) Status

Characteristic		HIV-positive n = 122		HIV-negative n = 47 470		*P* value
Age, median years (IQR)		56	(49, 62)	74	(60, 84)	<.001
Age group, n (%)	<40	7/120	(5.8)	2576/46 926	(5.5)	<.001
	40–49	26/120	(21.7)	3245/46 926	(6.9)	
	50–59	48/120	(40.0)	5945/46 926	(12.7)	
	60–69	26/120	(21.7)	7272/46 926	(15.5)	
	≥70	13/120	(10.8)	27 888/46 926	(59.4)	
Female, n (%)		41/121	(33.9)	20 302/47 303	(42.9)	.05
Ethnicity, n (%)	White	51/112	(45.5)	35 539/42 208	(84.2)	<.001
	Black	48/112	(42.9)	1475/42 208	(3.5)	
	Asian	1/112	(0.9)	2249/42 208	(5.3)	
	Other	12/112	(10.7)	2945/42 208	(7.0)	
Smoking, n (%)	Never	65/94	(69.2)	17 396/30 379	(57.3)	.004
	Former	18/94	(19.2)	10 634/30 379	(35.0)	
	Current	11/94	(11.7)	2340/30 379	(7.7)	
Comorbidities, median number (IQR)		1	(1, 2)	2	(1, 3)	<.001
Comorbidities, n (%)	None	31/122	(25.4)	9679/46 742	(20.7)	<.001
	1	50/122	(41.0)	13 544/46 742	(29.0)	
	2	28/122	(23.0)	11 529/46 742	(24.7)	
	≥3	13/122	(10.7)	11 990/46 742	(25.7)	
Type of comorbidities, n (%)	Chronic cardiac disease	20/117	(17.1)	14 620/45 054	(32.5)	<.001
	Chronic pulmonary disease^a^	13/120	(10.8)	8055/44 918	(17.9)	.04
	Asthma	12/116	(10.3)	6239/44 758	(13.9)	.26
	Chronic renal disease	21/116	(18.1)	7874/44 728	(17.6)	.89
	Diabetes, no complications	16/117	(13.7)	7783/43 862	(17.7)	.25
	Diabetes, with complications	9/117	(7.7)	3308/43 587	(7.6)	.97
	Obesity	19/112	(17.0)	4597/40 458	(11.4)	.06
	Chronic neurological disorder	8/116	(6.9)	5588/44 478	(12.6)	.07
	Dementia	3/118	(2.5)	7464/44 554	(16.8)	<.001
	Mild liver disease	3/118	(2.5)	632/44 228	(1.4)	.24
	Moderate/severe liver disease	6/118	(5.1)	861/44 281	(1.9)	.01
	Malignancy	4/118	(3.4)	4598/44 359	(10.4)	.009
	Chronic hematological disease	4/118	(3.4)	1931/44 311	(4.4)	.82
	Rheumatological disease	6/118	(5.1)	4874/44 183	(11.0)	.04
	Malnutrition	5/112	(4.5)	1133/41 853	(2.7)	.23

Abbreviation: IQR, interquartile range.

^a^Excludes asthma.

**Table 2.** Presenting Symptoms and Observations, Stratified by Human Immunodeficiency Virus (HIV) Status

Symptoms and Observations		HIV-positive n = 122		HIV-negative n = 47 470		*P* value
Presenting symptoms, n (%)	Fever	99/120	(82.5)	30 650/47 085	(65.1)	<.001
	Myalgia	28/104	(26.9)	6351/34 839	(18.2)	.02
	Headache	18/96	(18.8)	3661/34 790	(10.5)	.009
	Cough	96/121	(79.3)	31 028/47 077	(65.9)	.002
	Dyspnea	88/121	(72.7)	32 141/47 043	(68.3)	.30
	Chest pain	25/109	(22.9)	5227/38 302	(13.7)	.005
	Sore throat	14/100	(14.0)	2806/34 294	(8.2)	.03
	Wheeze	6/102	(5.9)	3293/36 241	(9.1)	.26
	Rhinorrhea	3/97	(3.1)	831/33 562	(2.5)	.52
	Diarrhea	28/108	(25.9)	7277/39 340	(18.5)	.05
	Nausea or vomiting	23/105	(21.9)	7650/39 560	(19.3)	.51
	Abdominal pain	13/104	(12.5)	4033/38 136	(10.6)	.52
	Fatigue	43/98	(43.9)	16 111/37 202	(43.3)	.91
	Asymptomatic	0/122	(0)	888/47 470	(1.9)	.18
Symptom group,^a^ n (%)	Systemic	108/121	(89.3)	32 267/47 119	(68.5)	<.001
	Respiratory	108/121	(88.5)	38 736/47 158	(82.1)	.04
	Gastrointestinal	45/111	(40.5)	13 444/41 311	(32.5)	.07
Symptom duration, median days (IQR)		5	(1, 9)	3	(0, 7)	.002
Symptom onset,^b^ n (%)	<3 days	114/122	(93.4)	41 285/47 101	(87.7)	.20
	3–7 days	1/122	(0.8)	1488/47 101	(3.2)	
	8–14 days	4/122	(3.3)	1603/47 101	(3.4)	
	>14 days	3/122	(2.5)	2725/47 101	(5.8)	
Presenting signs	Temperature, median °C (IQR)	37.8	(36.9, 38.6)	37.3	(36.6, 38.1)	.004
	Fever ≥37.8 °C, n (%)	60/117	(51.3)	16 447/45 458	(36.2)	.001
	HR, median beats/min (IQR)	96	(81, 110)	90	(78, 105)	.004
	Tachycardia,^c^ n (%)	52/117	(44.4)	15 076/45 432	(33.2)	.01
	RR, median breaths/min (IQR)	20	(18, 27)	21	(18, 26)	.97
	Tachypnea,^d^ n (%)	55/114	(48.3)	23 306/45 209	(51.6)	.48
	Hypoxia^e^/on oxygen, n (%)	56/115	(48.7)	23 971/45 242	(53.0)	036
	Infiltrates visible on CXR, n (%)	49/74	(66.2)	19 065/30 566	(62.4)	.50
	Systolic BP, median mmHg (IQR)	130	(118, 145)	130	(114, 147)	.78
	Diastolic BP, median mmHg (IQR)	80	(68, 86)	74	(65, 84)	.007

Abbreviations: BP, blood pressure; COVID-19, coronavirus disease 2019; CXR, chest X-ray; HR, heart rate; IQR, interquartile range; RR, respiratory rate.

^a^Systemic symptoms: ≥1 of fever, myalgia or headache; respiratory symptoms: ≥1 of cough, dyspnea, chest pain, sore throat, wheeze; gastrointestinal symptoms: ≥1 of: diarrhea, nausea, vomiting, or abdominal pain.

^b^Based on the onset of symptoms relative to the date of admission, COVID-19 acquisition was classed as community (<3 days), indeterminate (3–7 days), probable hospital (8–14 days), and definite hospital (>14 days).

^c^Defined as HR > 100 beats/min.

^d^Defined as RR > 20 breaths/min.

^e^Defined as SpO2 < 94% on air.

**Table 3.** Presenting Laboratory Parameters, Stratified by Human Immunodeficiency Virus (HIV) Status

Laboratory Parameter		HIV-positive n = 122		HIV-negative n = 47 470		*P* value
Hemoglobin, median g/L (IQR)		130	(117, 145)	129	(113, 143)	.56
Anemia,^a^ n (%)		39/107	(36.5)	15 570/40 092	(38.8)	.61
WBC, median count × 10^9^/L (IQR)		6.6	(4.8, 9.1)	7.4	(5.4, 10.4)	.01
Lymphocytes, median count × 10^9^/L (IQR)		1.0	(0.8, 1.5)	0.9	(0.6, 1.3)	<.001
Lymphopenia,^b^ n (%)		51/108	(47.2)	23 014/39 759	(57.9)	.03
Platelets, median count × 10^6^/L (IQR)		197	(150, 258)	217	(164, 286)	.01
Thrombocytopenia,^c^ n (%)		26/105	(24.8)	7440/39 739	(18.7)	.11
Prothrombin time, median s (IQR)		13.6	(11.0, 15.0)	13.2	(11.8, 15.0)	.58
Creatinine, median µmol/L (IQR)		89	(72, 134)	86	(67, 121)	.26
eGFR,^d^ median mL/min/1.73m^2^ (IQR)		75	(52, 101)	73	(48, 97)	.37
eGFR mL/min/1.73m^2^, n (%)	≥60	68/100	(68.0)	24 783/38 821	(63.8)	.15
	30–59	21/100	(21.0)	9786/38 821	(25.2)	
	15–29	4/100	(4.0)	2846/38 821	(7.3)	
	<15	7/100	(7.0)	1406/38 821	(3.6)	
ALT, median U/L (IQR)		28	(19, 46)	26	(17, 43)	.16
ALT > 40 U/L, n (%)		28/89	(31.5)	8458/30 478	(27.8)	.44
Glucose, median mmol/L IQR)		6.8	(5.8, 10.3)	6.8	(5.8, 8.9)	.44
Hyperglycemia,^e^ n (%)		11/54	(20.4)	2903/19 541	(14.9)	.26
C-reactive protein, median mg/L (IQR)		107	(51, 200)	83	(36, 157)	.02

Abbreviations: ALT, alanine transaminase; eGFR, estimated glomerular filtration rate; IQR, interquartile range; WBC, white blood cells.

^a^Defined as hemoglobin < 130 g/L in males and < 115 g/L in females.

^b^Defined as lymphocyte count < 1.0 × 10^9^/L.

^c^Defined as platelet count < 150 × 10^6^/L.

^d^Based on the Modification of Diet in Renal Disease (MDRD) formula where eGFR (mL/min/1.73 m^2^) = 175 × (Scr/88.4)-1.154 × (Age)-0.203 × (0.742 if female) × (1.212 if Black ethnicity).

^e^Defined as glucose >11 mmol/L.

### COVID-19 Outcomes

**Figure 3.**
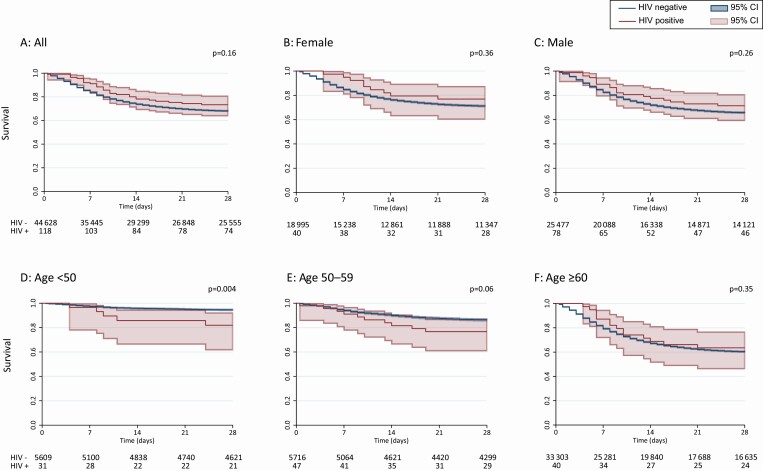
Kaplan-Meier survival plots stratified by HIV status. (*A*) All, (*B*) female, (*C*) male, (*D*) <50 years, (*E*) 50–59 years, (*F*) >60 years. *P* values are log-rank tests. Abbreviation: HIV, human immunodeficiency virus.

After adjustment for sex, ethnicity, age, baseline date, indeterminate/probable hospital acquisition of COVID-19, and 10 comorbidities, the odds of admission to critical care were similar regardless of HIV status (odds ratio 1.22; 95% confidence interval [CI] .80–1.87; *P* = .35) (Supplementary Table 3). By day 28 (Supplementary Table 4), 30 (24.6%) PWH were known to have died compared with 13 969 (29.4%) of the HIV-negative group; the cumulative incidence of day-28 mortality was 26.7% versus 32.1%, respectively (*P* = .16 Figure 3A). Whereas findings were similar in men and women (Figure 3B, C), univariate stratification for age revealed higher mortality among PWH in the younger age group (Figure 3D–F). Among participants under 60 years of age, mortality was 21.3% in HIV-positive patients versus 9.6% in HIV-negative patients (*P* < .001).

In the unadjusted analysis (Table 4), the cumulative hazard of day-28 mortality was lower in PWH (HR 0.77, 95% CI .54–1.11; *P* = .17). Results did not change after adjusting for sex or ethnicity. In contrast, adjustment for age resulted in a change in the direction of the association (adjusted HR 1.47, 95% CI 1.01–2.14; *P* = .05). Results were similar after adjustment for sex, ethnicity, age, baseline date, indeterminate/probable hospital acquisition of COVID-19 and 10 comorbidities, and following additional adjustment for disease severity at presentation. When restricted to people <60 years, the analysis yielded an adjusted HR of 2.87 (95% CI 1.70–4.86; *P* < .001). Sensitivity analyses showed consistent results (Supplementary Table 5). In particular, censoring follow-up on the day of discharge, including definite hospital-acquired COVID-19, using symptom onset as baseline and excluding PWH lacking an ART record did not significantly alter the model. A separate logistic regression model with a binary variable of day-14 mortality and a competing risk regression model with discharge as a competing risk for mortality also showed increased odds of mortality in the HIV-positive group.

**Table 4.** Cox Proportional Hazards Model of the Association Between Human Immunodeficiency Virus (HIV) Status and Day-28 Mortality

HIV-positive Versus HIV-negative	Hazard Ratio	95% CI	*P* value
Unadjusted	0.77	.54–1.11	.17
Adjusted for sex	0.76	.53–1.10	.15
Adjusted for ethnicity	0.88	.60–1.29	.52
Adjusted for age	1.47	1.01–2.14	.05
Adjusted for age and sex	1.45	1.00–2.12	.05
Adjusted for sex, ethnicity, age, baseline date, and indeterminate/probable hospital acquisition of COVID-19	1.49	1.01–2.20	.04
Adjusted for sex, ethnicity, age, baseline date, indeterminate/probable hospital acquisition of COVID-19, and 10 comorbidities^a^	1.50	1.02–2.22	.04
Adjusted for sex, ethnicity, age, baseline date, indeterminate/probable hospital acquisition of COVID-19, 10 comorbidities^a^, and hypoxia/receiving oxygen at presentation^b^	1.69	1.15–2.48	.008
Adjusted for sex, ethnicity, age, baseline date, indeterminate/probable hospital acquisition of COVID-19, 10 comorbidities^a^ and hypoxia/receiving oxygen at presentation^b^ among individuals aged <60 years	2.87	1.70–4.86	<.001

Abbreviations: CI, confidence interval; COVID-19, coronavirus disease 2019.

^a^The model adjusted separately for the following comorbidities: chronic cardiac disease, chronic pulmonary disease, chronic renal disease, diabetes, obesity, chronic neurological disorder, dementia. liver disease, malignancy, and chronic hematological disease.

^b^Hypoxia was defined as SpO2 < 94% on air; a record of hypoxia or receiving oxygen at presentation was used as an indicator of disease severity.

Among PWH, relative to patients who survived by day 28, patients who died were older and had a higher prevalence of diabetes and obesity and were less likely to have a record of ART (Table 5 and Supplementary Tables 6 and 7).

**Table 5.** Characteristics of Patients With Human Immunodeficiency Virus (HIV), Stratified by Outcome at Day 28, Selected Variables^a^

Characteristic		Died n = 30		Alive n = 92		*P* value
Age, median years (IQR)		58	(55, 70)	55	(49, 61)	.01
Age group, n (%)	<40	1/28	(3.6)	6/92	(6.5)	.12
	40–49	4/28	(13.8)	22/92	(23.9)	
	50–59	10/28	(37.9)	38/92	(41.3)	
	60–69	6/28	(20.7)	20/92	(21.7)	
	≥70	7/28	(24.1)	6/92	(6.5)	
ART recorded, n (%)		25/30	(80.7)	87/92	(94.6)	.07
Type of comorbidities, n (%)	Chronic pulmonary disease^b^	1/29	(3.5)	12/91	(13.2)	.19
	Diabetes, with complications	5/30	(16.7)	4/87	(4.6)	.03
	Obesity	8/28	(28.6)	11/84	(13.1)	.06
Presenting symptoms, n (%)	Cough	26/29	(89.7)	70/92	(76.1)	.19
	Diarrhea	8/26	(30.8)	20/82	(24.4)	.52
Symptom group, n (%)	Respiratory^c^	28/29	(96.6)	80/92	(87.0)	.19
Presenting signs	HR, median beats/min (IQR)	106	(95, 121)	92	(80, 108)	.003
	Tachycardia,^d^ n (%)	16/27	(59.3)	36/90	(40.0)	.08
	RR, median breaths/min (IQR)	26	(20, 30)	20	(18, 24)	.006
	Tachypnea,^e^ n (%)	18/27	(66.7)	37/87	(42.5)	.03
	Hypoxia^f^/on oxygen, n (%)	22/28	(78.6)	34/87	(39.1)	<.001
Laboratory parameters	WBC, median count × 10^9^/L (IQR)	8.0	(5.4, 11.8)	5.6	(4.6, 8.7)	.02
	eGFR,^g^ median ml/min/1.73m^2^ (IQR)	67	(50, 88)	77	(55, 107)	.13
	Glucose, median mmol/L (IQR)	10.4	(6.4, 13.2)	6.4	(5.8, 8.3)	.02
	Hyperglycemia,^h^ n (%)	6/15	(40.0)	5/39	(12.8)	.05
	C-reactive protein, median mg/L (IQR)	187	(97, 252)	92	(40, 157)	.001
Interventions, n (%)	Oxygen therapy during admission	22/30	(73.3)	54/87	(62.1)	.27
	Critical care admission	20/30	(66.7)	19/92	(20.7)	<.001
	Noninvasive ventilation	12/27	(44.4)	16/87	(18.4)	.006
	Invasive ventilation	13/29	(44.8)	6/87	(6.9)	<.001

Abbreviations: ART, antiretroviral therapy; BP, blood pressure; eGFR, estimated glomerular filtration rate; HR, heart rate; IQR, interquartile range; RR, respiratory rate; WBC, white blood cells.

^a^A full list of demographic and clinical characteristics is shown in Supplementary Table 6.

^b^Excludes asthma.

^c^Respiratory symptoms: ≥1 of cough, dyspnea, chest pain, sore throat, wheeze.

^d^Defined as HR > 100 beats/min.

^e^Defined as RR > 20 breaths/min.

^f^Defined as SpO2 < 94% on air.

^g^Based on the Modification of Diet in Renal Disease (MDRD) formula where eGFR (mL/min/1.73 m^2^) = 175 × (Scr/88.4)-1.154 × (Age)-0.203 × (0.742 if female) × (1.212 if Black ethnicity).

^h^Defined as glucose >11 mmol/L.

## DISCUSSION

### Principal Findings

This study found evidence suggesting an age-adjusted 47% increased risk of day-28 mortality among PWH hospitalized with COVID-19 compared to HIV-negative individuals in the same data set. Among people aged <60 years, HIV-positive status more than doubled the risk of mortality after adjusting for sex, ethnicity, age, baseline date, 10 separate comorbidities, and disease severity at presentation (as indicated by a record of hypoxia or receiving oxygen therapy). The latter adjustment considered that doctors may be more likely to admit HIV-positive adults with COVID-19 despite less severe symptoms.

The influence of age, sex, and ethnicity on COVID-19 outcomes is currently debated [1, 2, 25]. PWH in our study were significantly younger than the HIV-negative group and adjusting for age changed the direction of the association between HIV status and day-28 mortality, indicating that age was a significant confounder in our analyses. Men were more prevalent in the HIV-positive group, which is consistent with the epidemiology of HIV infection in the United Kingdom, where men represent just over two thirds of the whole population with HIV [26]. People of Black ethnicity comprised 42% of the HIV-positive group in this analysis relative to ~26% among PWH in the UK population [26]. Adjustment for sex or ethnicity alone did not impact our relative hazard estimates.

Although there is a recognized interplay between HIV and comorbidities, neither omitting the adjustment for comorbidities nor adjusting for separate comorbidities modified the association. PWH had fewer comorbidities, notably lower prevalence of chronic pulmonary disease and malignancies, and this is largely a function of their younger age. HIV-positive people who died were older and were more likely to suffer from obesity and diabetes with complications than those who survived to discharge. Similar trends have been seen in the general population [1, 25]. Although these observations highlight the importance of obesity and diabetes as cofactors, the increased risk of COVID-19 related mortality in PWH was not merely due to the presence of promoting comorbidities. It should be highlighted that we did not take into consideration differences in the control of comorbidities between the 2 groups.

### Comparison With Other Studies

Evidence about the interplay between HIV and COVID-19 is not entirely consistent [8, 9, 13–23]. A case-control study from New York compared 88 PWH, all of whom were receiving ART, and 405 HIV-negative controls matched by age, sex, ethnicity, and calendar week of infection [21]. The study found no difference in the outcomes of COVID-19 related hospitalization after adjusting for demographics, chronic obstructive pulmonary disease, smoking, and baseline ferritin and white blood cell count. There are important differences in the 2 study populations. Participants in the New York study had a median age of 61 years (interquartile range [IQR] 54–67), whereas we found excess mortality in HIV-positive people aged <60 years. Whereas malignancies were recorded less commonly in our cohort (3% vs 10%), prevalence of obesity was higher (17% vs 11%). It is also noteworthy that mortality in the overall ISARIC CCP-UK population was higher than that reported in the New York study [21] and other countries [1]. Consistent with our findings, there are preliminary data that HIV-positive status was associated with increased hazard of mortality (adjusted HR 2.75) in South Africa [23]. Although the analysis did not account for history of tuberculosis, obesity, and socioeconomic status, it is noteworthy that HIV suppression on ART did not alter the mortality risk.

### Strengths and Limitations of This Study

A key strength of this study was the ability to perform a direct comparison of people with and without HIV in the same data set, and to account for age, sex, ethnicity, and key comorbidities.

The data for this study were collected during the first peak of the UK COVID-19 epidemic, and there are missing data, including 2742 patients with missing HIV status, who were excluded from the analysis. We also excluded 10 people initially recorded as having HIV but whose HIV status could not be confirmed; this group was similar to the HIV-negative group, with a median age of 81, suggesting that their initial HIV record was incorrect. Thus, these missing data are unlikely to affect the results.

Our focus was on the effect of HIV-positive status on day-28 mortality among people hospitalized with COVID-19. We did not address risk factors for a COVID-19 diagnosis or hospitalization among PWH, or the suggested modulating role of certain antiretroviral agents [9, 10]. We also lacked data to adjust for deprivation or socioeconomic status. Our analysis cannot provide evidence of the role of HIV related parameters on outcomes of COVID-19 related hospitalization, as we did not have details of the ART history, plasma HIV-1 RNA load, CD4 cell count, and history of HIV related disease. HIV-positive people who died were less likely to have ART recorded than those who survived at day 28. This raises the possibility that some of the patients who died were not receiving ART, although this cannot be stated with certainty. In the United Kingdom, 93% of the 103 000 people estimated to have HIV infection have been diagnosed; of these, 97% receive ART with excellent virological suppression and only a small subset (~3%) is either not engaged with care or experiences problems with virological control despite ART [26]. Only a few PWH in our cohort had *Pneumocystis jirovecii* prophylaxis recorded in their admission medications, suggesting that the likelihood of PWH in our study being severely immunosuppressed was overall low.

Despite effective ART and normalized CD4 cell counts, a subset of PWH continue to experience immune activation, inflammation, and a procoagulatory state [7], which may modulate the risk of COVID-19 related morbidity and mortality [27, 28]. Persistent immune dysfunction may be the consequence of advanced immunocompromise prior to the start of ART, as defined by a low nadir CD4 cell count and inverted CD4:CD8 ratio. In the United Kingdom, 43% of people newly diagnosed with HIV in 2018 had a CD4 count <350 cells/mm^3^, a threshold indicative of significant immunosuppression [26, 29]. Furthermore, current guidelines about starting ART at the time to diagnosis were implemented relatively recently, whereas in the past ART initiation in the United Kingdom was deferred until the CD4 count had declined below thresholds of initially 200, then 350, and subsequently 500 cells/mm^3^ [6, 29]. Thus, many older PWH in the United Kingdom (and worldwide) will have experienced years of uncontrolled HIV replication prior to commencing treatment and may also have received earlier regimens of suboptimal efficacy, with potentially lasting effects on immune function [29, 30]. Immunological studies will be required to confirm these hypotheses.

In conclusion, our data are limited by the relatively small number of people with HIV included in the study, and the findings should be interpreted with caution. Nonetheless, after careful considerations, our analysis of the outcomes of patients hospitalized with COVID-19 in the United Kingdom shows an increased risk of day-28 mortality due to HIV-positive status. As the pandemic continues to spread, including in areas of increased HIV prevalence, it is important to record the HIV status of people hospitalized with COVID-19 and gather further data to corroborate our findings and confirm the population-specific determinants of outcomes. Meanwhile, emphasis for PWH should be placed on early HIV diagnosis, prompt ART initiation, and optimized screening for and control of comorbidities, including obesity and diabetes.

## Supplementary Data

Supplementary materials are available at *Clinical Infectious Diseases* online. Consisting of data provided by the authors to benefit the reader, the posted materials are not copyedited and are the sole responsibility of the authors, so questions or comments should be addressed to the corresponding author.

ciaa1605_suppl_Supplementary_Materials_1

## Notes

***Acknowledgments***. This work uses data provided by patients and collected by the National Health Service (NHS) as part of their care and support #DataSavesLives. The authors are extremely grateful to the 2648 frontline NHS clinical and research staff and volunteer medical students, who collected these data in challenging circumstances; and the generosity of the participants and their families for their individual contributions in these difficult times. They also acknowledge the support of Jeremy J. Farrar, Nahoko Shindo, Devika Dixit, Nipunie Rajapakse, Lyndsey Castle, Martha Buckley, Debbie Malden, Katherine Newell, Kwame O’Neill, Emmanuelle Denis, Claire Petersen, Scott Mullaney, Sue MacFarlane, Nicole Maziere, Julien Martinez, Oslem Dincarslan, and Annette Lake.

***Financial support.*** The work was supported by grants from: the National Institute of Health Research (award CO-CIN-01); the Medical Research Council (grant MC_PC_19059); the National Institute of Health Research Health Protection Research Units (HPRU) in (i) Emerging and Zoonotic Infections (NIHR200907) at the University of Liverpool in partnership with Public Health England (PHE) and in collaboration with the Liverpool School of Tropical Medicine and the University of Oxford, and (ii) Blood Borne and Sexually Transmitted Infections at University College London (UCL) in partnership with PHE and in collaboration with the London School of Hygiene and Tropical Medicine; the Wellcome Trust and the Department for International Development (215091/Z/18/Z), and the Bill and Melinda Gates Foundation (OPP1209135). A. S. is supported by a National Institute of Health Research Academic Clinical Lectureship at the University of Liverpool. L. T. is supported by the Wellcome Trust (grant number 205228/Z/16/Z). The Liverpool Experimental Cancer Medicine Centre provided infrastructure support for this research (C18616/A25153). The views expressed are those of the authors and not necessarily those of the DHSC, DID, NIHR, MRC, Wellcome Trust, or PHE.

***Potential conflicts of interest.*** All authors declare support from the National Institute for Health Research (NIHR), the Medical Research Council (MRC), the NIHR HPRU in Emerging and Zoonotic Infections at the University of Liverpool, and PHE, Wellcome Trust, Department for International Development (DID), the Bill and Melinda Gates Foundation, Liverpool Experimental Cancer Medicine Centre, and Department of Health and Social Care (DHSC) for the submitted work. A. M. G. reports personal fees from Roche Pharma Research & Early Development (pRED), consulting honoraria from Gilead, Janssen, and ViiV Healthcare, and research funding from Roche pRED, Gilead, Janssen and ViiV Healthcare outside the submitted work. G. V. reports research funding from ViiV Healthcare outside of the submitted work presented in this article. C. A. S. reports personal fees from Gilead Sciences and ViiV Healthcare for participation in Data Safety and Monitoring Boards, membership of Advisory Boards and for preparation of educational materials outside the submitted work. P. J. M. O. reports personal fees from consultancies for Janssen Scientific Advisory Board and from the European Respiratory Society for filming webcast on influenza and pneumonia vaccines; grants from MRC, MRC Global Challenge Research Fund (Human Infection Challenge Vaccine [HIC-Vac] Network), EU RSV Consortium in Europe (RESCEU), MRC/GSK EMINENT Network, NIHR Biomedical Research Centre, MRC/GSK, Wellcome Trust (Wellcome Trust Translation Fun Co), NIHR (HPRU in Respiratory Infection), UKRI MRC COVID-19 Rapid Response Call (COVID-19 ISARIC—Coronavirus Clinical Characterisation Consortium ISARIC-4C) and is an NIHR senior investigator outside the submitted work; his role as President of the British Society for Immunology was unpaid but travel and accommodation at some meetings was provided by the Society. L. T. reports grants from Wellcome Trust, grants from UKRI, grants from Health Protection Research Unit in Emerging & Zoonotic Infections, University of Liverpool, during the conduct of the study. J. K. B. reports grants from MRC during the conduct of this study. M. G. S. reports grants from DHSC NIHR UK, MRC UK, and HPRU in Emerging and Zoonotic Infections during the conduct of the study; minority ownership of Integrum Scientific LLC (Greensboro, NC, USA) outside the submitted work. All other authors report no potential conflicts. All authors have submitted the ICMJE Form for Disclosure of Potential Conflicts of Interest. Conflicts that the editors consider relevant to the content of the manuscript have been disclosed.

***Author contributions.*** A. M. G. designed the study concept, reviewed all aspects of the data analysis and interpretation (of which she is the guarantor), contributed to the writing of the manuscript, and performed the final review of the manuscript. A. J. S. performed the data analysis and contributed to the data interpretation and the writing of the manuscript. S. H. K. performed the initial literature search and contributed to the conceptualization, data analysis and interpretation, and the writing of the manuscript. M. C., L. W., and S. C. contributed to conceptualization and review of the manuscript. G. V. contributed to data analysis and interpretation and the writing of the manuscript. A. D., E. M. H., and L. T. contributed to the ISARIC CCP-UK study design and data collection and reviewed the manuscript. P. J. M. O., ISARIC CCP-UK Co-Lead investigator, sourced funding, contributed to the ISARIC CCP-UK study design and data collection, and reviewed the manuscript. J. K. B., ISARIC CCP-UK Consortium lead investigator, sourced funding, contributed to the ISARIC CCP-UK study design and data collection and reviewed the manuscript. C. A. S. advised on all aspects of the conceptualization and data analysis and interpretation, and contributed to the writing and final review of the manuscript. M. G. S., ISARIC CCP-UK Protocol Chief Investigator and guarantor of the data, sourced permissions and funding, contributed to the ISARIC CCP-UK study design and data collection, and reviewed the manuscript. A. M. G. and M. G. S. accept full responsibility for the conduct of the study, had full access to the data, and controlled the decision to publish. A. M. G. attests that all listed authors meet authorship criteria and that no others meeting the criteria have been omitted.

**ISARIC 4C investigators**

Consortium Lead Investigator: J Kenneth Baillie, Chief Investigator Malcolm G Semple.

Co-Lead Investigator: Peter JM Openshaw. ISARIC Clinical Coordinator: Gail Carson.

Co-Investigators: Beatrice Alex, Benjamin Bach, Wendy S Barclay, Debby Bogaert, Meera Chand, Graham S Cooke, Annemarie B Docherty, Jake Dunning, Ana da Silva Filipe, Tom Fletcher, Christopher A Green, Ewen M Harrison, Julian A Hiscox, Antonia Ying Wai Ho, Peter W Horby, Samreen Ijaz, Saye Khoo, Paul Klenerman, Andrew Law, Wei Shen Lim, Alexander, J Mentzer, Laura Merson, Alison M Meynert, Mahdad Noursadeghi, Shona C Moore, Massimo Palmarini, William A Paxton, Georgios Pollakis, Nicholas Price, Andrew Rambaut, David L Robertson, Clark D Russell, Vanessa Sancho-Shimizu, Janet T Scott, Louise Sigfrid, Tom Solomon, Shiranee Sriskandan, David Stuart, Charlotte Summers, Richard S Tedder, Emma C Thomson, Ryan S Thwaites, Lance CW Turtle, Maria Zambon. Project Managers Hayley Hardwick, Chloe Donohue, Ruth Lyons, Wilna Oosthuyzen, Fiona Griffiths.

Data Analysts: Lisa Norman, Riinu Pius, Tom M Drake, Cameron J Fairfield, Stephen Knight, Kenneth A Mclean, Derek Murphy, Catherine A Shaw.

Data and Information System Manager: Jo Dalton, Michelle Girvan, Egle Saviciute, Stephanie Roberts, Janet Harrison, Laura Marsh, Marie Connor, Sophie Halpin, Clare Jackson, Carrol Gamble.

Data integration and presentation: Gary Leeming, Andrew Law, Ross Hendry.

Material Management: William Greenhalf, Victoria Shaw, Sarah McDonald.

Outbreak Laboratory Volunteers: Katie A. Ahmed, Jane A Armstrong, Milton Ashworth, Innocent G Asiimwe, Siddharth Bakshi, Samantha L Barlow, Laura Booth, Benjamin Brennan, Katie Bullock, Benjamin WA Catterall, Jordan J Clark, Emily A Clarke, Sarah Cole, Louise Cooper, Helen Cox, Christopher Davis, Oslem Dincarslan, Chris Dunn, Philip Dyer, Angela Elliott, Anthony Evans, Lewis WS Fisher, Terry Foster, Isabel Garcia-Dorival, Willliam Greenhalf, Philip Gunning, Catherine Hartley, Antonia Ho, Rebecca L Jensen, Christopher B Jones, Trevor R Jones, Shadia Khandaker, Katharine King, Robyn T. Kiy, Chrysa Koukorava, Annette Lake, Suzannah Lant, Diane Latawiec, L Lavelle-Langham, Daniella Lefteri, Lauren Lett, Lucia A Livoti, Maria Mancini, Sarah McDonald, Laurence McEvoy, John McLauchlan, Soeren Metelmann, Nahida S Miah, Joanna Middleton, Joyce Mitchell, Shona C Moore, Ellen G Murphy, Rebekah Penrice-Randal, Jack Pilgrim, Tessa Prince, Will Reynolds, P. Matthew Ridley, Debby Sales, Victoria E Shaw, Rebecca K Shears, Benjamin Small, Krishanthi S Subramaniam, Agnieska Szemiel, Aislynn Taggart, Jolanta Tanianis-Hughes, Jordan Thomas, Erwan Trochu, Libby van Tonder, Eve Wilcock, J. Eunice Zhang.

Local Principal Investigators: Kayode Adeniji, Daniel Agranoff, Ken Agwuh, Dhiraj Ail, Ana Alegria, Brian Angus, Abdul Ashish, Dougal Atkinson, Shahedal Bari, Gavin Barlow, Stella Barnass, Nicholas Barrett, Christopher Bassford, David Baxter, Michael Beadsworth, Jolanta Bernatoniene, John Berridge, Nicola Best, Pieter Bothma, David Brealey, Robin Brittain-Long, Naomi Bulteel, Tom Burden, Andrew Burtenshaw, Vikki Caruth, David Chadwick, Duncan Chambler, Nigel Chee, Jenny Child, Srikanth Chukkambotla, Tom Clark, Paul Collini, Catherine Cosgrove, Jason Cupitt, Maria-Teresa Cutino-Moguel, Paul Dark, Chris Dawson, Samir Dervisevic, Phil Donnison, Sam Douthwaite, Ingrid DuRand, Ahilanadan Dushianthan, Tristan Dyer, Cariad Evans, Chi Eziefula, Chrisopher Fegan, Adam Finn, Duncan Fullerton, Sanjeev Garg, Sanjeev Garg, Atul Garg, Jo Godden, Arthur Goldsmith, Clive Graham, Elaine Hardy, Stuart Hartshorn, Daniel Harvey, Peter Havalda, Daniel B Hawcutt, Maria Hobrok, Luke Hodgson, Anita Holme, Anil Hormis, Michael Jacobs, Susan Jain, Paul Jennings, Agilan Kaliappan, Vidya Kasipandian, Stephen Kegg, Michael Kelsey, Jason Kendall, Caroline Kerrison, Ian Kerslake, Oliver Koch, Gouri Koduri, George Koshy, Shondipon Laha, Susan Larkin, Tamas Leiner, Patrick Lillie, James Limb, Vanessa Linnett, Jeff Little, Michael MacMahon, Emily MacNaughton, Ravish Mankregod, Huw Masson, Elijah Matovu, Katherine McCullough, Ruth McEwen, Manjula Meda, Gary Mills, Jane Minton, Mariyam Mirfenderesky, Kavya Mohandas, Quen Mok, James Moon, Elinoor Moore, Patrick Morgan, Craig Morris, Katherine Mortimore, Samuel Moses, Mbiye Mpenge, Rohinton Mulla, Michael Murphy, Megan Nagel, Thapas Nagarajan, Mark Nelson, Igor Otahal, Mark Pais, Selva Panchatsharam, Hassan Paraiso, Brij Patel, Justin Pepperell, Mark Peters, Mandeep Phull, Stefania Pintus, Jagtur Singh Pooni, Frank Post, David Price, Rachel Prout, Nikolas Rae, Henrik Reschreiter, Tim Reynolds, Neil Richardson, Mark Roberts, Devender Roberts, Alistair Rose, Guy Rousseau, Brendan Ryan, Taranprit Saluja, Aarti Shah, Prad Shanmuga, Anil Sharma, Anna Shawcross, Jeremy Sizer, Richard Smith, Catherine Snelson, Nick Spittle, Nikki Staines, Tom Stambach, Richard Stewart, Pradeep Subudhi, Tamas Szakmany, Kate Tatham, Jo Thomas, Chris Thompson, Robert Thompson, Ascanio Tridente, Darell Tupper-Carey, Mary Twagira, Andrew Ustianowski, Nick Vallotton, Lisa Vincent-Smith, Shico Visuvanathan, Alan Vuylsteke, Sam Waddy, Rachel Wake, Andrew Walden, Ingeborg Welters, Tony Whitehouse, Paul Whittaker, Ashley Whittington, Meme Wijesinghe, Martin Williams, Lawrence Wilson, Sarah Wilson, Stephen Winchester, Martin Wiselka, Adam Wolverson, Daniel G Wooton, Andrew Workman, Bryan Yates, Peter Young.

**CHASE investigators**

Chief Investigator: Anna Mara Geretti.

Co-Investigators: Caroline Sabin, Sophie Kelly, Alexander Stockdale, Giovanni Villa, Laura Waters, Muge Cevik, Simon Collins, Daniel Bradshaw, Alison Brown, Nicky Connor, Valerie Delpech, Saye Khoo, Tamyo Mbisa, Chloe Orkin, Ann Sullivan.

**Data availability**

The CO-CIN data were collated by ISARIC4C Investigators. ISARIC4C welcomes applications for data and material access through our Independent Data and Material Access Committee (https://isaric4c.net). ISARIC4C has a public facing website and twitter account @CCPUKstudy. We are engaging with print and internet press, television, radio, news, and documentary makers. These findings have been distributed through community groups including UK-CAB, HIV i-Base and via the British HIV Association.

**Role of funder/sponsor**

The funders had no role in the design and conduct of the study; collection, management, analysis, and interpretation of the data; preparation, review, or approval of the manuscript; and decision to submit the manuscript for publication. Professor Geretti (the manuscript’s guarantor) affirms that the manuscript is an honest, accurate, and transparent account of the study being reported; that no important aspects of the study have been omitted; and that any discrepancies from the study as planned (and, if relevant, registered) have been explained.

**For queries related to the ISARIC CCP-UK study**

Prof Malcolm G Semple, PhD, FRCPE, FRCPCH, Institute of Infection, University of Liverpool, 8 West Derby St, Liverpool L69 7BE, UK; Email: M.G.Semple@liverpool.ac.uk; twitter @ProfCalumSemple; Telephone: +44 795 833 5337 ORCID 0000-0001-9700-0418.
