# Supplementary material for: Outcomes of Coronavirus Disease 2019 (COVID-19) Related Hospitalization Among People With Human Immunodeficiency Virus (HIV) in the ISARIC World Health Organization (WHO) Clinical Characterization Protocol (UK): A Prospective Observational Study
Source: Clin Infect Dis. 2020 Oct 23;73(7):e2095–106. doi: 10.1093/cid/ciaa1605 (PMC7665382; doi:10.1093/cid/ciaa1605)
Supplement: ciaa1605_suppl_Supplementary_Materials_1 [file ciaa1605_suppl_supplementary_materials_1.docx]

**Supplementary Table 1.** Characteristics of participants excluded from the analysis (due to missing date of admission or symptom onset, date of admission or symptom onset after 4^th^ June 2020, missing HIV status, or unconfirmed HIV-positive status) relative to those included.

| Characteristic | | Excluded | | | | | | | |  | Included | | | | |
| --- | --- | --- | --- | --- | --- | --- | --- | --- | --- | --- | --- | --- | --- | --- | --- |
|  |  | Missing date  of admission or symptom onset  n=2,694 | | Symptom onset >4^th^ June 2020  n=303 | | Missing HIV status  n=2,742 | | Unable to confirm  HIV-positive status  n=10 | |  | People with HIV  n=122 | | | HIV-negative group  n=47,470 | |
| Age, median years (IQR) | | 73 (55, 83) | | 75 (61, 86) | | 73 (61, 85) | | 81 (62, 86) | |  | 56 (49, 62) | | | 74 (60, 84) | |
| Age group,  n (%) | <40 | 145/1,330 | (10.9) | 28/301 | (9.3) | 151/2,662 | (5.7) | 0/10 | (0) |  | 7/120 | (5.8) | 2,576/46,926 | | (5.5) |
|  | 40-49 | 106/1,330 | (8.0) | 19/301 | (6.3) | 176/2,662 | (6.6) | 1/10 | (10.0) |  | 26/120 | (21.7) | 3,245/46,926 | | (6.9) |
|  | 50-59 | 157/1,330 | (11.8) | 26/301 | (8.6) | 321/2,662 | (12.1) | 0/10 | (0) |  | 48/120 | (40.0) | 5,945/46,926 | | (12.7) |
|  | 60-69 | 172/1,330 | (12.9) | 46/301 | (15.3) | 377/2,662 | (14.2) | 2/10 | (20.0) |  | 26/120 | (21.7) | 7,272/46,926 | | (15.5) |
|  | ≥70 | 750/1,330 | (56.4) | 182/301 | (60.5) | 1,637/2,662 | (61.5) | 7/10 | (70.0) |  | 13/120 | (10.8) | 27,888/46,926 | | (59.4) |
| Female, n (%) | | 579/1,278 | (45.3) | 155/303 | (51.2) | 1,160/2,685 | (43.2) | 4/10 | (40.0) |  | 42/122 | (34.4) | 20,302/47,303 | | (42.9) |
| Ethnicity,  n (%) | White | 866/1,020 | (84.3) | 237/280 | (84.6) | 1,567/2,030 | (77.2) | 5/8 | (62.5) |  | 51/112 | (45.5) | 35,539/42,208 | | (84.2) |
|  | Black | 25/1,020 | (2.6) | 7/280 | (2.5) | 69/2,030 | (3.4) | 2/8 | (25.0) |  | 48/112 | (42.9) | 1,475/42,208 | | (3.5) |
|  | Asian | 64/1,020 | (6.5) | 21/280 | (7.5) | 228/2,030 | (11.2) | 0/8 | (0) |  | 1/112 | (0.9) | 2,249/42,208 | | (5.3) |
|  | Other | 65/1,020 | (6.7) | 15/280 | (5.4) | 166/2,030 | (8.2) | 1/8 | (12.5) |  | 12/112 | (10.7) | 2,945/42,208 | | (7.0) |
| Outcomes,  n (%) | Critical care | 15/205 | (7.3) | 42/260 | (16.2) | 125/983 | (12.7) | 0/10 | (0) |  | 39/122 | (32.0) | 6,706/46,497 | | (14.4) |
|  | Invasive ventilation | 11/194 | (5.7) | 11/242 | (4.6) | 33/545 | (6.1) | 0/9 | (0) |  | 19/116 | (16.4) | 3,999/45,252 | | (8.8) |
|  | Died | 102/303 | (33.7) | 12/79 | (15.2) | 541/1,096 | (49.4) | 2/9 | (22.2) |  | 30/111 | (27.0) | 14,555/43,015 | | (33.8) |

Abbreviations: IQR, Interquartile range.

**Supplementary Table 2.**  Regional distribution of study participants with HIV compared to the total United Kingdom population of PWH accessing care in 2018^a^

| Region | Study participants with HIV, n (%) | | People receiving HIV  care in 2018, n (%) | |
| --- | --- | --- | --- | --- |
| London | 44/122 | (35.8) | 36,689/96,135 | (38.2) |
| Midlands & East of England | 25/122 | (20.3) | 19,387/96,135 | (20.2) |
| North of England | 26/122 | (21.1) | 16,500/96,135 | (17.2) |
| South of England | 23/122 | (18.7) | 15,426/96,135 | (16.0) |
| Scotland | 5/122 | (4.1) | 4,799/96,135 | (5.0) |
| Wales | 0 | (0) | 2,204/96,135 | (2.3) |
| Northern Ireland | 0 | (0) | 1,130/96,135 | (1.2) |

^a^Data from Public Health England^26^

**Supplementary Table 3.** Binomial logistic regression analysis of the likelihood of admission to critical care according to HIV status

| People with HIV versus HIV-negative group | Odds ratio | 95% CI | P value |
| --- | --- | --- | --- |
| Unadjusted | 2.79 | 1.90-4.08 | <0.001 |
| Adjusted for sex | 2.69 | 1.83-3.96 | <0.001 |
| Adjusted for age | 1.70 | 1.14-2.52 | 0.009 |
| Adjusted for sex, age, ethnicity, start date, indeterminate/probable hospital acquisition of COVID-19, and 10 comorbidities^a^ | 1.22 | 0.80-1.87 | 0.35 |

^a^The final model adjusted for the following comorbidities: chronic cardiac disease, chronic pulmonary disease, chronic renal disease, diabetes, obesity, chronic neurological disorder, dementia, liver disease, malignancy, and chronic haematological disease.

Abbreviations: CI, Confidence interval.

**Supplementary Table 4.** COVID-19 outcomes according to HIV status and follow up time^a^

|  | People with HIV n=122 | | HIV-negative group  n=47,470 | |
| --- | --- | --- | --- | --- |
| Outcome at 14 days | | | | |
| Death | 25 | (20.5) | 11,563 | (24.4) |
| Discharged alive | 61 | (50.0) | 19,254 | (40.6) |
| Remaining in hospital | 21 | (17.2) | 11,298 | (23.8) |
| Transfer to other facility | 4 | (3.3) | 1,252 | (2.6) |
| Unknown outcome | 11 | (9.0) | 4,103 | (8.6) |
| Outcome at 28 days | | | | |
| Death | 30 | (24.6) | 13,969 | (29.4) |
| Discharged alive | 70 | (57.4) | 23,598 | (49.7) |
| Remaining in hospital | 7 | (5.7) | 3,710 | (7.8) |
| Transfer to other facility | 4 | (3.3) | 1,798 | (3.8) |
| Unknown outcome | 11 | (9.0) | 4,395 | (9.3) |
| Outcome at last known follow up | | | | |
| Death | 30 | (24.6) | 14,555 | (30.7) |
| Discharged alive | 75 | (61.5) | 25,373 | (53.5) |
| Transfer to other facility | 4 | (3.3) | 2,100 | (4.4) |
| Remaining in hospital | 2 | (1.6) | 987 | (2.1) |
| Unknown outcome | 11 | (9.0) | 4,455 | (9.4) |

**^a^**Unknown outcome: an outcome had not been recorded at the time of analysis

**Supplementary Table 5.** Sensitivity analyses investigating the association between HIV status and 28-day mortality

| Analysis |  | Hazard ratio,  odds ratio or sub hazard ratio | 95% CI | P value |
| --- | --- | --- | --- | --- |
| 1 | Model 1, Primary Cox proportional hazard model of day-28 mortality, people with HIV versus comparators, adjusted for sex, ethnicity age, baseline date, indeterminate/probable hospital acquisition of COVID-19, and 10 comorbidities^a^ | HR 1.50 | 1.02-2.22 | 0.04 |
| 2 | Model 1, Adjusted for hypoxia or receiving oxygen therapy at presentation | HR 1.69 | 1.15-2.48 | 0.008 |
| 3 | Model 1, Sensitivity analysis 1: Follow up right-censored at discharge for patients discharged alive (instead of at day 28) | HR: 1.52 | 1.02-2.25 | 0.04 |
| 4 | Model 1, Sensitivity analysis 2: Including definite hospital acquired COVID-19^b^ | HR: 1.55 | 1.06-2.26 | 0.02 |
| 5 | Model 2, Sensitivity analysis 3: Using symptom onset date as baseline date for all patients | HR: 1.53 | 1.02-2.31 | 0.04 |
| 6 | Model 2, Sensitivity analysis 4: Excluding PWH lacking a record of ART | HR: 1.35 | 0.90-2.04 | 0.15 |
| 7 | Model 2, Sensitivity analysis 5: Including only those with a positive SARS-CoV-2 PCR result recorded in the CRF | HR 1.66 | 1.11-2.49 | 0.01 |
| 8 | Sensitivity analysis 6: Cox proportional hazards model including propensity score for HIV status^c^ | HR: 1.43 | 0.98-2.08 | 0.06 |
| 9 | Sensitivity analysis 7: Cox proportional hazards model including propensity score for HIV status and hypoxia or need for oxygen therapy at presentation^c^ | HR 1.59 | 1.10-2.29 | 0.01 |
| 10 | Sensitivity analysis 8: Binary logistic regression model of day-14 mortality, people with HIV versus comparators, adjusted for sex, ethnicity age, baseline date, indeterminate/probable hospital acquisition of COVID-19, and 10 comorbidities^a^ | OR: 1.64 | 1.00-2.70 | 0.05 |
| 11 | Sensitivity analysis 9: Competing risks regression model with discharge before 28 days included as a competing risk for mortality: people with HIV versus comparators, adjusted for sex, ethnicity age, baseline date, indeterminate/probable hospital acquisition of COVID-19, and 10 comorbidities^a^ | SHR: 1.50 | 1.02 – 2.20 | 0.04 |

^a^The model adjusted for the following comorbidities: chronic cardiac disease, chronic pulmonary disease, chronic renal disease, diabetes, obesity, chronic neurological disorder, dementia. liver disease, malignancy, and chronic haematological disease. ^b^Based on onset of symptoms >14 days after admission. ^c^The propensity score for HIV status was derived from a logistic regression model including the following covariates: sex, ethnicity, age (in quadratic form), indeterminate/probable hospital acquisition of COVID-19, smoking status, baseline date, and ten comorbidities^a^.

**Supplementary Table 6.** Characteristics of patients with HIV, stratified by outcome at day 28, selected variables

| Characteristic | | Died  n=30 | | Alive  n=92 | | P-value |
| --- | --- | --- | --- | --- | --- | --- |
| Age, median years (IQR) | | 58 | (53, 70) | 55 | (49, 61) | 0.01 |
| Age group,  n (%) | <40 | 1/28 | (3.6) | 6/92 | (6.5) | 0.12 |
|  | 40-49 | 4/28 | (14.3) | 22/92 | (23.9) |  |
|  | 50-59 | 10/28 | (35.7) | 38/92 | (41.3) |  |
|  | 60-69 | 6/28 | (21.4) | 20/92 | (21.7) |  |
|  | ≥70 | 7/28 | (25.0) | 6/92 | (6.5) |  |
| Female, n (%) | | 9/30 | (30.0) | 33/92 | (35.9) | 0.56 |
| Ethnicity,  n (%) | White | 13/28 | (46.4) | 38/84 | (45.2) | 0.47 |
|  | Black | 10/28 | (35.7) | 38/84 | (45.2) |  |
|  | Asian | 0/28 | (0) | 1/84 | (1.2) |  |
|  | Other | 5/28 | (17.9) | 7/84 | (8.3) |  |
| Smoking,  n (%) | Never | 16/26 | (61.5) | 49/68 | (72.1) | 0.21 |
|  | Former | 8/26 | (30.8) | 10/68 | (14.7) |  |
|  | Current | 2/26 | (7.7) | 9/68 | (13.2) |  |
| ART recorded, n (%) |  | 25/30 | (83.3) | 87/92 | (94.6) | 0.07 |
| Number of comorbidities, median (IQR) |  | 1 | (1,2) | 1 | (0,2) | 0.41 |
| Comorbidities,  n (%) | None | 4/30 | (13.3) | 27/92 | (29.4) | 0.28 |
|  | 1 | 16/30 | (53.3) | 34/92 | (37.0) |  |
|  | 2 | 7/30 | (23.3) | 21/92 | (22.8) |  |
|  | $\geq$3 | 3/30 | (10.0) | 10/92 | (10.9) |  |
| Type of comorbidities, n (%) | Chronic cardiac disease | 4/29 | (13.8) | 16/88 | (18.2) | 0.78 |
|  | Chronic pulmonary disease^a^ | 1/29 | (3.5) | 12/91 | (13.2) | 0.19 |
|  | Asthma | 4/28 | (14.3) | 8/88 | (9.1) | 0.48 |
|  | Chronic renal disease | 4/28 | (14.3) | 17/88 | (19.3) | 0.78 |
|  | Diabetes, no complications | 5/30 | (16.7) | 11/87 | (12.6) | 0.55 |
|  | Diabetes, with complications | 5/30 | (16.7) | 4/87 | (4.6) | 0.03 |
|  | Obesity | 8/28 | (28.6) | 11/84 | (13.1) | 0.06 |
|  | Chronic neurological disorder | 2/29 | (6.9) | 6/87 | (6.9) | 1.0 |
|  | Dementia | 0/29 | (0) | 3/89 | (3.4) | 1.0 |
|  | Mild liver disease | 0/30 | (0) | 3/88 | (3.4) | 0.57 |
|  | Moderate/severe liver disease | 1/30 | (3.3) | 5/88 | (5.7) | 1.0 |
|  | Malignancy | 1/30 | (3.3) | 3/88 | (3.4) | 1.0 |
|  | Chronic haematological disease | 1/30 | (3.3) | 3/88 | (3.4) | 1.0 |
|  | Rheumatological disease | 2/30 | (6.7) | 4/88 | (4.6) | 0.65 |
|  | Malnutrition | 1/28 | (3.6) | 4/84 | (4.8) | 1.0 |
| Presenting symptoms,  n (%) | Fever | 24/28 | (85.7) | 75/92 | (81.5) | 0.78 |
|  | Myalgia | 5/24 | (20.8) | 23/80 | (28.8) | 0.60 |
|  | Headache | 3/23 | (13.0) | 15/73 | (20.6) | 0.55 |
|  | Cough | 26/29 | (89.7) | 70/92 | (76.1) | 0.19 |
|  | Dyspnoea | 21/29 | (72.4) | 67/92 | (72.8) | 0.97 |
|  | Chest pain | 6/25 | (24.0) | 19/84 | (22.6) | 0.89 |
|  | Sore throat | 1/22 | (4.6) | 13/78 | (16.7) | 0.29 |
|  | Wheeze | 3/25 | (12.0) | 3/77 | (3.9) | 0.16 |
|  | Rhinorrhoea | 1/23 | (4.4) | 2/74 | (2.7) | 0.56 |
|  | Diarrhoea | 8/26 | (30.8) | 20/82 | (24.4) | 0.52 |
|  | Nausea or vomiting | 4/26 | (15.4) | 19/79 | (24.1) | 0.42 |
|  | Abdominal pain | 2/25 | (8.0) | 11/79 | (13.9) | 0.73 |
|  | Fatigue | 9/23 | (39.1) | 34/75 | (45.3) | 0.60 |
| Symptom group^b^,  n (%) | Systemic | 26/29 | (89.7) | 82/92 | (89.1) | 1.0 |
|  | Respiratory | 28/29 | (96.6) | 80/92 | (87.0) | 0.19 |
|  | Gastrointestinal | 10/27 | (37.0) | 35/84 | (41.7) | 0.67 |
| Symptom duration, median days (IQR) | | 5 | (1, 9) | 5 | (1, 9) | 0.71 |
| Symptom onset relative to admission ^c^,  n (%) | <3 days | 28/30 | (93.3) | 86/92 | (93.5) | 1.0 |
|  | 3-7 days | 0/30 | (0) | 1/92 | (1.1) |  |
|  | 8-14 days | 1/30 | (3.3) | 3/92 | (3.3) |  |
|  | >14 days | 1/30 | (3.3) | 2/92 | (2.2) |  |
| Presenting signs | Temperature, median °C (IQR) | 37.7 | (36.9, 38.6) | 37.8 | (36.9, 38.6) | 0.73 |
|  | Fever ≥37.8 °C, n (%) | 13/27 | (48.2) | 47/90 | (52.2) | 0.83 |
|  | HR, median beats/min (IQR) | 106 | (95, 121) | 92 | (80, 108) | 0.003 |
|  | Tachycardia^d^, n (%) | 16/27 | (59.3) | 36/90 | (40.0) | 0.08 |
|  | RR, median breaths/min (IQR) | 26 | (20, 30) | 20 | (18, 24) | 0.006 |
|  | Tachypnoea^e^, n (%) | 18/27 | (66.7) | 37/87 | (42.5) | 0.03 |
|  | Hypoxia^f^, n (%) | 22/28 | (78.6) | 34/87 | (39.1) | <0.001 |
|  | Infiltrates visible on CXR, n (%) | 12/17 | (70.6) | 37/57 | (64.9) | 0.78 |
|  | Systolic BP, median mmHg (IQR) | 131 | (114, 146) | 130 | (118, 145) | 0.81 |
|  | Diastolic BP, median mmHg (IQR) | 75 | (65, 83) | 81 | (69, 88) | 0.09 |
| Laboratory parameters | Haemoglobin, median g/L (IQR) | 128 | (114, 141) | 132 | (118,146) | 0.50 |
|  | Anaemia, n (%) | 12/27 | (44.4) | 27/80 | (33.8) | 0.32 |
|  | WBC, median count x109/L (IQR) | 8.0 | (5.4, 11.8) | 5.6 | (4.6, 8.7) | 0.02 |
|  | Lymphocytes, median count x10^9^/L (IQR) | 0.9 | (0.7, 1.3) | 1.0 | (0.8, 1.5) | 0.41 |
|  | Lymphopenia, n (%) | 15/28 | (53.6) | 36/80 | (45.0) | 0.43 |
|  | Platelets, median count x106/L (IQR) | 192 | (150, 236) | 200 | (147, 263) | 0.73 |
|  | Thrombocytopenia, n (%) | 6/27 | (22.2) | 20/78 | (25.6) | 0.72 |
|  | Prothrombin time, median sec (IQR) | 14.5 | (11.4, 15.0) | 13.2 | (11.0, 15.0) | 0.33 |
|  | Creatinine, median µmol/L (IQR) | 87 | (70, 156) | 90 | (72, 132) | 0.71 |
|  | eGFRd, median ml/min/1.73m2 (IQR) | 67 | (50, 88) | 77 | (55, 107) | 0.13 |
|  | ALT, median U/L (IQR) | 34 | (20, 46) | 27 | (18, 46) | 0.51 |
|  | ALT >40 U/L, n (%) | 8/24 | (33.3) | 20/65 | (30.8) | 0.82 |
|  | Glucose, median mmol/L (IQR) | 10.4 | (6.4, 13.2) | 6.4 | (5.8, 8.3) | 0.02 |
|  | Hyperglycaemia, n (%) | 6/15 | (40.0) | 5/39 | (12.8) | 0.05 |
|  | C-reactive protein, median mg/L (IQR) | 187 | (97, 252) | 92 | (40, 157) | 0.001 |
| Interventions, n (%) | Oxygen therapy during admission | 22/30 | (73.3) | 54/87 | (62.1) | 0.27 |
|  | Critical care admission | 20/30 | (66.7) | 19/92 | (20.7) | <0.001 |
|  | Non-invasive ventilation | 12/27 | (44.4) | 16/87 | (18.4) | 0.006 |
|  | Invasive ventilation | 13/29 | (44.8) | 6/87 | (6.9) | <0.001 |

^a^ Excluding asthma, ^b^Systemic symptoms: ≥1 of fever, myalgia or headache; Respiratory symptoms: ≥1 of cough, dyspnoea, chest pain, sore throat, wheeze; Gastrointestinal symptoms: ≥1 of: Diarrhoea, nausea, vomiting or abdominal pain.  ^c^Based on the onset of symptoms relative to the date of admission, COVID-19 acquisition was classed as community onset (<3 days), indeterminate (3-7 days), probable hospital (8-14 days), and definite hospital onset (>14 days). ^d^Defined as HR >100 beats/min. ^e^Defined as RR >20 breaths/min. ^f^Defined as SpO2 <94% on air or receiving oxygen therapy at presentation;

**Supplementary Table 7.** Cox proportional hazard model for day-28 mortality among people with HIV

| **Comparison** | **Univariate**  **hazard ratio** | **95% CI** | **P value** |
| --- | --- | --- | --- |
| Age, per year | 1.05 | (1.01- 1.09) | 0.005 |
| Sex, male vs female | 1.41 | (0.65-3.08) | 0.39 |
| Chronic heart disease | 0.76 | (0.27-2.18) | 0.61 |
| Asthma | 1.38 | (0.48-3.95) | 0.55 |
| Pulmonary disease (excluding asthma) | 0.26 | (0.04-1.91) | 0.19 |
| Diabetes | 2.18 | (1.02-4.66) | 0.04 |
| Obesity | 2.89 | (1.28-6.53) | 0.01 |
| ART recorded | 0.39 | (0.15-1.01) | 0.05 |

Abbreviation: ART antiretroviral therapy.
